# A Poultry Subclinical Necrotic Enteritis Disease Model Based on Natural *Clostridium perfringens* Uptake

**DOI:** 10.3389/fphys.2022.788592

**Published:** 2022-06-08

**Authors:** Wanwei He, Emanuele C. Goes, Jeremy Wakaruk, Daniel R. Barreda, Douglas R. Korver

**Affiliations:** ^1^ Department of Biological Sciences, University of Alberta, Edmonton, AB, Canada; ^2^ Department of Agricultural, Food and Nutritional Science, University of Alberta, Edmonton, AB, Canada

**Keywords:** broiler chicken, *Clostridium perfringens*, environment, natural infection model, necrotic enteritis

## Abstract

Necrotic enteritis (NE) in poultry is an opportunistic infection caused by *Clostridium perfringens*. Well-known as a multifactorial disease, NE development is under the influence of a wide range of environmental risk factors that promote the proliferation of pathogenic *C. perfringens* at the expense of nonpathogenic strains. Current *in vivo* NE challenge models typically incorporate pre-exposure to disease risk factors, in combination with exogenous *C. perfringens* inoculation. Our goal was to enhance current models using a natural uptake of *C. perfringens* from the barn environment to produce a subclinical infection. We incorporated access to litter, coccidial exposure (either 10× or 15× of the manufacturer-recommended Coccivac B52 *Eimeria* vaccine challenge; provided unspecified doses of *E. acervulina, E. mivati, E. tenella*, and two strains of *E. maxima*), feed composition, and feed withdrawal stress, and achieved the commonly observed NE infection peak at 3 weeks post-hatch. NE severity was evaluated based on gut lesion pathology, clinical signs, and mortality rate. Under cage-reared conditions, 15× coccidial vaccine-challenged birds showed overall NE lesion prevalence that was 8-fold higher than 10× coccidial vaccine-challenged birds. NE-associated mortality was observed only in a floor-reared flock after a 15× coccidial vaccine challenge.

## 1 Introduction

Necrotic enteritis (NE) is an economically important infectious disease for the global poultry sector, causing an annual loss of US $6 billion worldwide (Wade and Keyburn, 2015). This is largely attributable to the costs of prophylactic and therapeutic medications and compromised growth performance. The causative bacterium, *Clostridium perfringens*, is ubiquitously distributed and comprises part of the gut microbiota of healthy chickens, with a high diversity of strains representing the total *C. perfringens* population (Engström et al., 2012; Yang et al., 2018; Kiu et al., 2019). The NE-causing strains are characterized by a capacity to produce necrotic enteritis toxin B (NetB) and possession of genes that function to enhance their proliferation, maintenance, and virulence, including antibiotic resistance genes, adhesins, catabolic enzymes, toxins, and bacteriocins (Bannam et al., 2011; Parreira et al., 2012; Freedman et al., 2015; Keyburn et al., 2008).

One key step in NE pathogenesis is the dominance of pathogenic *C. perfringens* in the gut flora over nonpathogenic strains, followed by profound expression of virulence factors. A number of risk factors promote pathogenic *C. perfringens* development, and hence NE infection. Exposure to coccidial parasites remains one of the best-studied factors due to the strong link between coccidiosis and NE disease (Stanley et al., 2014). Coccidiosis-induced epithelial extracellular matrix disruption, plasma protein leakage, and mucus production provide extra selective advantages for pathogenic *C. perfringens* which possess a stronger binding ability and mucolytic activity than nonpathogenic strains (Collier et al., 2008; Martin and Smyth, 2010). Diet components also constitute relevant key risk factors associated with NE development. Feeds rich in water-soluble non-starch polysaccharides, such as wheat-based diets, increase digesta viscosity, prolong transit time, and promote pathogen retention (Annett et al., 2002; Shojadoost et al., 2012). Increased NE occurrence is also associated with poor husbandry management, such as food deprivation, inadequate hygiene routines, and overcrowding (Hofacre et al., 2019).

Given the multifactorial nature of NE, the production of experimental infections similar to field conditions is known to be challenging. Typically, induced NE outbreaks should occur around week three post-hatch, reflecting the timing when animals in the field are most at risk (Williams, 2005; Moore, 2016). Additionally, the model should yield a high incidence of necrotic lesions without severe mortality in the flock (Dierick et al., 2021). This is relevant to the field situation where subclinical infections are more common and account for larger economic loss compared to the clinical form of NE (Wade & Keyburn, 2015). Efforts over the past decade suggest that concentrated live coccidial vaccines in combination with multiple dosages of *C. perfringens* culture result in NE infections that fulfill these criteria (Gholamiandehkordi et al., 2007; Dierick et al., 2021). Recent NE studies often adopt this dual-infection approach concurrently with the application of dietary and management risk factors (Dierick et al., 2019; Lee et al., 2013; M'Sadeq et al., 2015; Onrust et al., 2018; Wilson et al., 2018).

**FIGURE 1:**
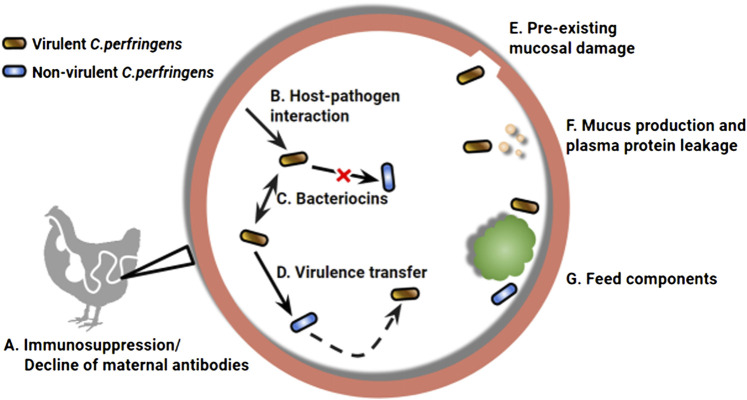
A wide range of environmental factors determines the manifestation of necrotic enteritis (NE) caused by *Clostridium perfringens*. Chickens are at higher risk to develop NE infection with impaired immune status (A), which can result from declining maternal antibodies and/or pre-exposure to immunosuppressive factors (Lee et al., 2011). The intestine environment also plays a role in virulence modulation of *C. perfringens*, the first example can be seen in the upregulated toxin expression when in close contact with epithelial cells (B) (Vidal et al., 2009). A complex microbiota environment, compared to a culture medium, can differentially regulate the virulence phenotype of the bacterium *via* inter and intra strain interactions through bacteriocin production (C) (Ohtani and Shimizu, 2015). Virulent strains are capable of producing bacteriocins that strongly inhibit non-virulent strains, as a mechanism in pathogen-commensal competition (Barbara et al., 2008; Timbermont et al., 2009). Meanwhile, non-virulent strains can inherit the virulence genes horizontally (D), leading to the emergence of new strains that are capable of causing NE (Lacey et al., 2017). Preexisting mucosal damage that exposes epithelial extracellular matrix (E), increased plasma protein leakage and mucus production (F) can provide an extra advantage for pathogenic *C perfringens*, which possess a stronger binding ability and mucolytic activity (Collier et al., 2008; Martin and Smyth, 2010). Diet components comprising part of the gut environment are also key risk factors associated with NE development (G). For example, feeds rich in water-soluble non-starch polysaccharides, such as a wheat-based diet, can increase digesta viscosity, prolong transit time, and promote pathogen retention (Annett et al., 2002; Shojadoost et al., 2012).

Induction of experimental NE using natural exposure to *C. perfringens* further mirrors conditions under which NE arises in commercial operations. This approach has gained prominence over the past decade (Abildgaard et al., 2010; Calik et al., 2019; Emami et al., 2019 and 2021; Fernando et al., 2011; Lovland et al., 2003; Palliyeguru et al., 2010). Compared to conventional models that drive infection *via* experimental application of *C. perfringens*, natural NE infection can A) simplify the disease challenge protocol, B) eliminate the variation between models caused by different bacterial culture conditions, challenge route, dosage, timing, and frequency, and C) develop subclinical infection most similar to the field condition. Importantly, *C. perfringens* undergoes a series of adaptations in response to fluctuation of the gut environment, which modify the disease-causing ability of this bacterium (Figure 1). Given the highly plastic phenotype that *C. perfringens* can display natural infection also overcomes deviations commonly associated with *in vitro* manipulation, including changes to colonization efficacy and toxin production (Parreira et al., 2016). Thus, the natural infection approach can better recapitulate the microbial loads and other relevant physiological factors that contribute to NE pathogenesis.

**FIGURE 2:**
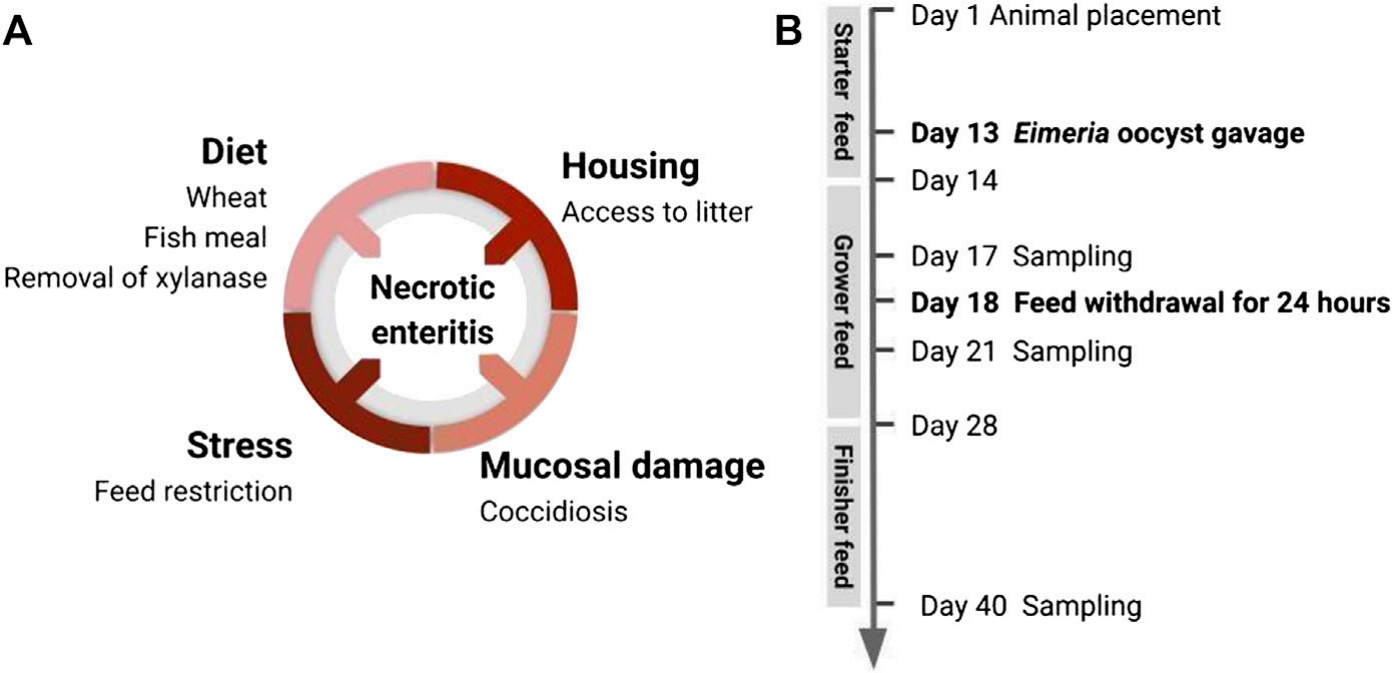
Induction of subclinical NE in broiler chicken by application of disease predisposing factors**. (A)** To promote natural development of the already-existing virulent *C perfringens* in the gut, this disease model incorporated multiple predisposing factors: housing condition, diet components, coccidiosis-induced mucosal damage, and stressor leading to alteration of gut environment. **(B)** Timeline of animal handling and sampling schedule.

Our objective was to validate a natural, subclinical NE challenge model. Aiming to optimize the current natural NE model, we incorporated a novel stressor, a 24-h feed withdrawal at day 18 post-hatch, apart from other commonly used risk factors for inducing experimental NE. This nutrient alteration in the gut lumen aims to disrupt the intestinal microbial community and promote the development of pathogenic *C. perfringens*. To examine whether this infection protocol induces subclinical NE in different rearing conditions, we challenged three experimental flocks with different housing types, diet regimens, and two levels of coccidial challenge intensity. Our results suggested timely application of stress factors (Figure 2) resulted in a consistent NE infection similar to the field situation, characterized by a high incidence of gut lesions in the flock with a low mortality rate.

## 2 Material and Methods

### 2.1 Animals and Natural Necrotic Enteritis Challenge

One-day-old Ross 708 broiler chicks were obtained from a local hatchery (Sofina Foods) and housed in the Poultry Research Center at the University of Alberta, Edmonton, Canada. The natural NE infection model was developed stepwise using a total of 752 animals from three experimental flocks. Animals in flocks 1 and 3 were randomly assigned to two dietary treatments to evaluate the impact of antibiotic removal on NE development (flock 1: antibiotic treatment with 21 cages of 8 birds, and drug-free treatment with 22 cages of 8 birds; flock 3: antibiotic treatment with 8 pens of 18 birds, drug-free treatment with 8 pens of 18 birds). Animals in flock 2 were used for evaluating the immunomodulation effect of β-glucan and were randomly assigned to three injection treatments (each with 5 cages of 8 birds). Flocks 1 and 2 were reared in Specht pullet cages (21 × 23.5 × 17.5 inches, Specht Canada Inc.), and flock 3 was housed in the floor pens (0.9 m × 1.4 m). All three flocks were treated with a natural NE challenge procedure but with different levels of coccidiosis challenge intensity (Table 3).

Birds from all three flocks were fed a wheat-based diet formulated to meet or exceed the management guide recommendations for all nutrients. The experimental diets were administered as a starter diet, grower diet, and finisher diet (Table 1). The diet composition for flock 3 was adjusted as part of an adaptation of the model to include a more practical commercial-type diet. Feed and water were provided ad libitum. Temperature and lighting were monitored daily and adjusted according to the Ross 708 guidelines (Aviagen, 2019). On day 13, a 10× (flock 1) or 15× (flocks 2 and 3) dose of the Coccivac-B52 vaccine (Merck Animal Health) containing live, sporulated *Eimeria* oocysts (*E. acervulina*, *E. mivati*, *E. tenella*, and two strains of *E. maxima* at unspecified doses) was administered through oral gavage. Each bird received 1 ml of vaccine diluted in distilled water. On day 18, the feed was withdrawn for 24 h with animals being closely monitored for health over the subsequent 3 days. Figure 2B shows the predisposing factors application timeline in the natural NE challenge model.

**TABLE 1:** Ingredient and calculated nutrient composition of experimental diets for birds during starter, grower, and finisher stages.

	Flocks 1 and 2			Flock 3		
	Starter	Grower	Finisher	Starter	Grower	Finisher
Ingredients (%)						
Canola meal	5	7.5	10	7.5	10	12
Fish meal	4	4	4	-	-	-
Soybean meal	24.05	17.62	11.75	27.96	22.44	19.38
Wheat	62.25	65.44	67.18	59.18	61.46	61.40
Limestone	0.92	0.78	0.66	1.18	1.03	0.93
Monocalcium phosphate	0.43	0.20	-	1.00	0.75	0.57
NaCl	0.30	0.30	0.30	0.27	0.26	0.26
l-Lysine	0.06	0.06	0.92	0.10	0.07	0.02
dl-Methionine	0.26	0.22	0.2	0.30	0.25	0.23
l-Threonine	0.05	0.03	0.01	0.05	0.01	-
Hy-D^®^ Premix^a^	0.05	0.05	0.05	0.05	0.05	0.05
Vitamin Mineral Premix^b^	0.5	0.5	0.5	0.5	0.5	0.5
Choline Chloride Premix^c^	0.05	0.05	0.05	0.05	0.05	0.05
Phytase^d^	0.01	0.01	0.01	0.01	0.01	0.01
Canola oil	1.62	2.79	3.92	1.86	3.12	4.63
Mycotoxin binder^e^	0.05	0.05	0.05	0.15	0.15	0.15
Xylanase^f^	—	—	—	0.05	0.05	0.05
Calculated nutrient composition						
Crude protein	25.5	23.62	22.65	25.2	23.58	22.73
ME, kcal/kg	3,000	3,100	3,200	3,000	3,100	3,200
Calcium	0.96	0.87	0.79	0.96	0.87	0.81
Available phosphorus	0.48	0.435	0.395	0.48	0.435	0.405

a Provided 69 µg 25-hydroxycholecalciferol per kg diet.

b Provided per kilogram of diet: vitamin A (retinyl acetate), 10,000 IU; cholecalciferol, 4,000 IU; vitamin E (DL-α-tocopheryl acetate), 50 IU; vitamin K, 4.0 mg; thiamine mononitrate (B_1_), 4.0 mg; riboflavin (B_2_), 10 mg; pyridoxine HCL (B_6_), 5.0 mg; vitamin B_12_ (cobalamin), 0.02 mg; d-pantothenic acid, 15 mg; folic acid, 0.2 mg; niacin, 65 mg; biotin, 1.65 mg; iodine (ethylenediamine dihydroiodide), 1.65 mg; Mn (MnSO4H2O), 120 mg; Cu, 20 mg; Zn, 100 mg, Se, 0.3 mg; Fe (FeSO_4_·7H_2_O), 800 mg.

c Provided 100 mg choline per kg of diet.

d Provided 500 FTU phytase per kg of diet (Phyzyme XP, Danisco Animal Nutrition, Marlborough, United Kingdom).

e Biomin II (Biomin Canada Inc. Mont-St-Hilaire, Québec, Canada).

f Econase XT, 25 (AB, Vista, Marlborough, United Kingdom) provided 80,000 BXU, of endo-1, 4-beta-xylanase activity per kg diet.

### 2.2 Sampling and Lesion Scoring

For experimental flocks 1 and 2, animals were randomly selected and examined for NE disease status on days 17, 21, and 40 (flock 1: *n* = 16, flock 2: *n* = 18). Flock 3 was sampled on day 21 and day 40 (*n* = 128). The NE-specific lesions in the small intestine were scored as described by Shojadoost et al. (2012) with some modifications. Animals were scored from 0 to 3 based on the following criteria:

0: No gross lesion;

1: Thin or friable walls, or diffuse superficial fibrin;

2: Focal necrosis or ulceration, or non-removable fibrin deposit;

3: Multifocal necrosis or ulceration, or nonremovable fibrin deposit.

Lesions more severe than score 3 were not observed.

### 2.3 Bacterial Quantification

#### 2.3.1 DNA Extraction and Purification

Cecal contents collected from birds in flocks 1 and 2 were kept at −20°C for *C. perfringens* quantification. 0.2 g of thawed cecal content was measured into a 2 ml tube with 0.3 g 0.1 mm diameter silica beads (Biospec). The cecal contents were washed with and resuspended in 1 ml of TN150 buffer (149 mM NaCl, 5.58 mM Tris-HCl, 4.38 mM Trometamol, and pH 8.0) followed by a 3 min bead-beating at 5,000 rpm (Mini BeadBeater, Fisher Scientific). After centrifugation at 14,600 g for 5 min, the supernatant was transferred to a new 2 ml microtube. The DNA was purified using the phenol and chloroform-isoamyl alcohol (24:1) method and precipitated with 100% ethanol at −20°C overnight. The DNA pellet was washed twice with 500 μL of 70% ethanol without disrupting the pellet and dissolved in 100 μL of Nuclease-free water. The concentration and quality of DNA were measured using an ND-1000 spectrophotometer (NanoDrop Technologies) at 260 and 280 nm.

#### 2.3.2 Quantitative Real-Time PCR (qRT-PCR)

The total *C. perfringens* population was quantified by qRT-PCR targeting the 16s rRNA gene (Table 2). Commercial *C. perfringens* genomic DNA was serially diluted 7-fold (using 1.35 × 10^7^ as a starting point) and included on each plate to generate a standard curve for the absolute quantification of the bacteria population.

**TABLE 2:** *Clostridium perfringens* qPCR targeted genes and primer sequences used in this study.

Target	Sense	Sequence	References
16S rRNA	Fw	GGG​TTT​CAA​CAC​CTC​CGT​G	AP017630.1
	Rv	GCA​AGG​GAT​GTC​AAG​TGT​AGG	
netB	Fw	TGA​TAC​CGC​TTC​ACA​TAA​AGG​TTG​G	Yang et al. (2018)
	Rv	ATA​AGT​TTC​AGG​CCA​TTT​CAT​TTT​TCC​G	

The qRT-PCR experiment was performed in QuantStudio™ 6 Flex System (Applied Biosystems) and data were analyzed with a QuantStudio rt-PCR Software v.1.3 (Applied Biosystems). Reactions of each sample were triplicated on a 96-well plate containing a 20 μL reaction mixture in each well (1 μL 50 ng/μL DNA template, 1 μL 25 pmol/μL of forward and reverse primers, 10 μL Fast SYBR Green Master Mix, and 7 μL Nuclease-free water). The amplification process started with initial denaturation at 95°C for 20 s followed by 40 cycles of annealing including 95°C for 3 s and 62°C for 30 s. As an indicator of amplification specificity, the melting curve of PCR products was generated by fluorescence collection during slow heating from 60 to 95°C with a rate of 0.05°C/s. The copy number of the target gene was calculated by the following formula as described by Li et al. (2009) and expressed as copies/g digesta. $DNA\;\left(number\;of\;molecules\right)=\frac{\left[6.02\times 10^{23}\left(\frac{molecules}{mol}\right)\times DNA\;amount\;\left(g\right)\right]}{\left[DNA\;length\left(bp\right)\times 660\left(\frac{\frac{g}{mol}}{bp}\right)\right]}$

To detect the presence of the netB gene in cecal digesta, the genomic DNA of a netB-positive strain (CA147, Arden Biotechnology Ltd., United Kingdom) was 7-fold serial diluted, as aforementioned, and included on each plate to generate a standard curve for the absolute quantification of the strains. Reactions of each sample containing a 20 μL reaction mixture were prepared as aforementioned. The amplification process started at 95°C for 20 s, followed by 40 cycles of annealing and elongation including 95°C for 3 s and 60°C for 30 s. The specific netB amplicon was differentiated from nonspecific products by the DNA melting curve. The copy number of the target genes (*C. perfringens* and netB-positive strains) were calculated according to Li et al. (2009) and expressed as copies/g digesta.

### 2.4 Intestine Histology

Following lesion scoring, a 3 cm intestinal section at the lesion site was collected and fixed in 10% formaldehyde for microscopic histology analysis. Fixed intestine tissue was dehydrated and embedded in paraffin wax, then sliced into 5 μm sections and stained with hematoxylin and eosin. The intestinal cross sections were examined under light microscopy. Images were collected by using a SeBaCam digital microscope camera with SeBaView software (Thermo Scientific).

### 2.5 Statistical Analysis

Statistical analyses were performed by the GraphPad Prism 8 software. The experimental unit was the individual bird. To understand the impact of the coccidial challenge on natural NE development, Fisher’s exact test was conducted to compare the lesion score between low- and high-coccidial challenged animals. A nonparametric Mann-Whitney test was used to compare the rank of lesion score between two coccidial challenge levels of the same age. T-tests were conducted to compare bacteria abundance between the age of days 17 and 21. *p* < 0.05 was defined as being statistically significant.

## 3 Results

### 3.1 Mortality and Clinical Signs

The two cage-reared flocks yielded similar mortality during the experiment period. The overall mortality (day 1–40) was 1.15% in flock 1 and 1.39% in flock 2 (Table 3). All mortalities occurred within the first week, prior to the coccidial vaccine dosing and feed withdrawal challenge. Birds did not show observable clinical signs, but bloody and mucous-containing feces were found after feed withdrawal, indicating the presence of diarrhea.

**TABLE 3:** Mortality and intestinal lesion prevalence in three experimental flocks with different flock sizes, housing types, and coccidiosis challenge intensities. Coccidial pre-exposure was incorporated in the NE disease model through oral gavage of live Eimeria oocysts using the Coccivac-B52 vaccine (Merck Animal Health).

	Flock 1	Flock 2	Flock 3
Flock size	344	120	288
Housing type	cage	cage	floor
Eimeria dosage^a^	10×	15×	15×
Overall mortality (%)	1.15%	1.39%	2.86%
Mortality (%), day 18–35	0	0	1.51%
Overall lesion prevalence	10.42%	85.19%	80.08%

a A concentrated Coccivac-B52 vaccine was applied at 10× (flock 1) or 15× (flocks 2 and 3) of the recommended dosage. Each bird received 1 ml of vaccine diluted in distilled water.

The floor-reared flock (flock 3) showed higher overall mortality at 3.82% compared to the cage-reared flocks (Table 3). Two mortality peaks were observed during the experiment period. The first peak occurred during week 1 and the second peak was found between weeks 3–5 after the 24-h feed withdrawal was applied. The second mortality peak was directly triggered by feed withdrawal on day 18, which also caused depression and decreased mobility in birds.

### 3.2 Detection and Quantification of *C. perfringens*

**FIGURE 3:**
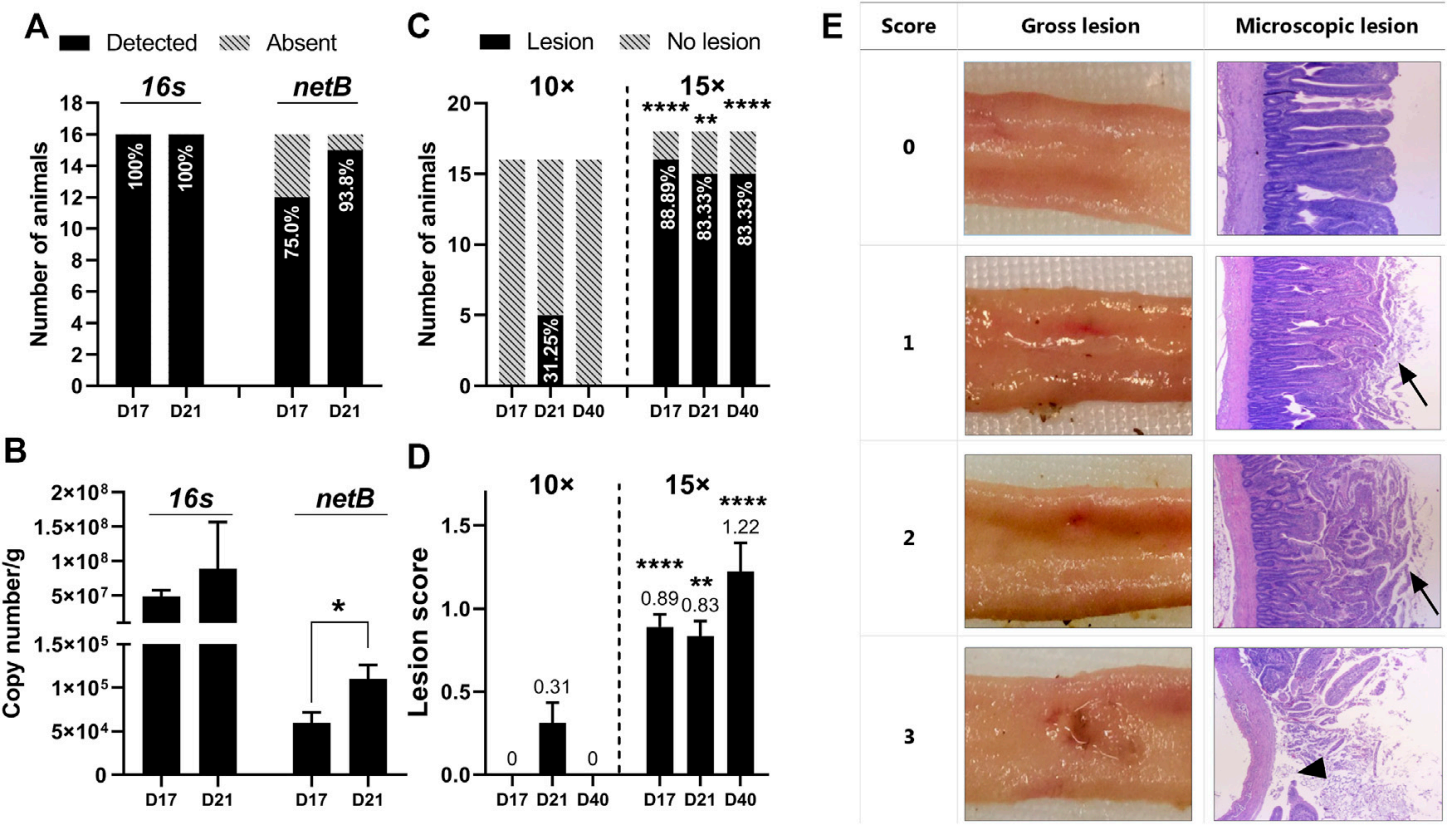
Quantification of *C* perfringens and intestine lesion confirmed induction of subclinical necrotic enteritis using the natural infection model **(A)** Detection of *C perfringens* 16s and netB gene in cecal contents by qRT-PCR. The percentages of animals detected with 16s or netB are plotted within bars. Data were collected from flock 1 (*n* = 16) **(B)** Abundance of 16s and netB gene expressed as copy number/g of cecal content. Data were collected from flock 1. T-tests were conducted to compare gene abundance between the age of days 17 and 21. The netB abundance on day 21 was significantly higher than on day 17 (*p* = 0.0242) **(C)** Intestine gross lesion prevalence in flock 1 (challenged with 10× concentrated coccidial vaccine) and flock 2 (challenged with 15× concentrate/d coccidial vaccine). The percentages of animals detected with gross lesions are plotted within bars. Fisher’s exact test was conducted to compare the difference between low and high coccidial challenged animals (D17: *p* < 0.0001, D21: *p* = 0.0045; D40: *p* < 0.0001) **(D)** Lesion scoring results from flock 1 and flock 2. A nonparametric Mann-Whitney test was used to compare the rank of lesion score between two coccidial challenge levels from the same age (D17: *p* < 0.0001, D21: *p* = 0.0045, D40: *p* < 0.0001) **(E)** Severity of NE-specific lesions was scored from 0 to 3 based on the intestine gross examination. The tissue at the lesion site was processed for the histology analysis. The original magnification of the images is ×25. The necrotic tissue was typically covered by a layer of mixed cellular debris (arrow). Sloughed mucosa leading to complete loss of villi (arrowhead) was observed in intestinal tissue with a score of 3.

Cecal total *C. perfringens* was quantified by qRT-PCR targeting the 16 s gene. In flock 1, all sampled birds were found to be *C. perfringens* positive regardless of age (Figure 3A). The presence of netB, the hallmark of NE-causing strains, was detected in 75.0% of the samples on day 17, before the feed withdrawal challenge, and increased to 93.8% on day 21. Correspondingly, netB abundance on day 21 was significantly higher than day 17 (*p* = 0.0242) (Figure 3B). This is consistent with our expectation that the 24-h feed withdrawal on day 18 further contributed to the propagation of the virulent strains within the flock. The observed *C. perfringens* density was relatively high with 16s abundance ranging from 10⁷ to 10⁹ copies per gram of cecal content (Figure 3B). A relatively lower netB abundance was observed at around 10⁶ copies per gram of digesta. Quantification of 16 s and netB copies showed no significant difference between day 17 and day 21 (*p* > 0.05), though day 21 tended to have a higher abundance of the examined genes. Flock 2 was reared in similar conditions as flock 1 but was challenged with a higher dosage of Eimeria oocysts on day 13. However, the higher coccidial challenge level did not increase bacteria detection rate or bacteria abundance (data not shown).

### 3.3 Gross Examination of Intestine Lesion

In our study, mild lesions (scores 1 and 2) were predominantly observed with a few birds scored with 3 (Figure 3E). Severe lesions (score greater than 3) was not observed in any of the flocks. A total of 48 birds were sampled in experiment flock 1. Only 5 birds (10.42%) had NE-specific lesions under the 10× coccidiosis vaccine challenge (Table 3), and all the lesion-positive animals were observed on day 21. With increased intensity of coccidial challenge at (15× vaccine dose), flock 2 (*n* = 54) and flock 3 (*n* = 256) showed a higher prevalence of birds with necrotic lesions (85.19 and 80.08%, respectively). Lesion prevalence in flocks reared in the wire-floored cage environment (flock 1 and 2) showed that coccidial challenge levels have a profound impact on NE lesion development (Figure 3C). The 15× dosage of Eimeria vaccine gavage led to prevalent lesion development in the flock as early as day 17 and the lesions were also present on day 40. The high-coccidial challenge flock showed increased lesion score compared to the low-challenge flock on day 17 (*p* < 0.0001), day 21 (*p* = 0.0045), and day 40 (*p* < 0.0001) (Figure 3D). The floor-rear flock (flock 3) with 15× coccidial challenge yielded consistently higher lesion prevalence on both sampling days as expected. The observed lesion prevalence was 75% (96/128) on day 21 and 93.75% (120/128) on day 40. Animals in flock 3 were not sampled on day 17.

### 3.4 Microscopic Examination of Intestine Lesion

Representative histopathological images of the intestinal section are shown in Figure 3E. Intestine tissue scored at 0 with no gross lesion and generally showed intact villus structure. However, examination under higher magnification revealed pathological changes including the presence of Eimeria oocysts, mildly dilated capillaries, and capillary hemorrhage. Tissue lesions that scored 1 and 2 generally showed similar microscopic appearance though distinguished changes were observed in gross examination. Under microscopic examination, the necrotic region showed hyperemia, villus fusion, and separation of epithelium from the lamina propria. The necrotic tissue was usually covered by adherent fibrin and cellular debris. These pathological alterations were also observed in lesioned tissue with a score of 3. Noticeably, sloughed mucosa leading to complete loss of villi was observed in certain areas within the lesioned tissue (Figure 3E).

## 4 Discussion

### 4.1 Confirmation of Necrotic Enteritis Development

Bacteria quantification together with the characteristic pathology of NE, such as clinical signs and gut lesions, is indicative of successful induction of NE disease (Williams et al., 2003; McReynolds et al., 2004; Palliyeguru et al., 2010). *C. perfringens* can be found in high populations in NE-affected animals ranging from 10⁶ ∼ 10⁹ CFU (Williams, 2005; McDevitt et al., 2006; Abildgaard et al., 2010). The *C. perfringens* population observed in our trial is consistent with those typically found in NE-affected animals. Interestingly, the netB gene was more prevalent on day 21 compared to day 17 (Figure 3A). This may be associated with the observed diarrhea on days 18–19 following the feed withdrawal challenge, which could indicate that the experimental conditions promoted the spreading of the netB-carrying strains in the flock.

As noted in previous studies, there may be a poor correlation between the number of *C. perfringens* organisms in the digesta and the incidence or severity of necrotic enteritis, especially in the subclinical form of the disease (Fernando et al., 2011). Subclinical NE is usually mild with no clinical signs or sudden increase in mortality (Fernando et al., 2011). Thus, gut lesions are considered to be a sensitive disease indicator compared to clinical signs and mortality (Williams et al., 2003). In this study, gut lesions were found in all three experimental flocks and were confirmed with microscopic examination (Figure 3E). Together, the observed bacterial load, clinical signs, and pathological changes suggest NE occurrence in the flocks with disease severity peaked during weeks 3 to 4.

### 4.2 Prevalence of Gut Lesion

Many conventional NE disease models have shown lesion incidence peaks at a certain age and declines as the animal approaches slaughter (Lovland et al., 2003; Gholamiandehkordi et al., 2007). Natural NE infection induced by reused litter material from a previous flock (Palliyeguru et al., 2010), high stocking density, and housing of birds on litter (Lovland et al., 2003; Fernando et al., 2011) have resulted in lesion prevalence ranging from 6.9 to 68.6%. However, to our knowledge, most studies conducted only one lesion examination during the rearing period. In the field conditions, subclinical NE-affected animals can be detected at slaughter with *C. perfringens*-associated lesions in the liver and gut (Lovland and Kaldhusdal, 1999; Johansson et al., 2010). This suggests experimental NE models with persisting lesion occurrence may better reflect NE cases in the field, where birds suffering from the subclinical disease are kept without being treated. In this study, we thus involved multiple gut examinations throughout the rearing period. Results suggested coccidial challenge has a profound influence on the development of gut NE lesions. Coccidial challenge intensity affects the occurrence and duration of gross lesions present in the NE-affected flocks. As noted by Stanley et al. (2014), *Eimeria* spp. caused significant changes in gut microbiota diversity and enabled *C. perfringens* to persist post challenge. *C. perfringens* inoculated in the absence of this predisposing factor fail to establish and maintain themselves in the gut flora.

### 4.3 Mortality

Epidemiology studies suggest that NE occurrence is associated with specific housing conditions, including access to litter and floor-type housing (Kaldhusdal et al., 2016; Goossens et al., 2020). Our data shows NE-related mortality was not observed in our cage-reared flocks even with a 15× coccidial challenge dosage. A sudden but minor increase in mortality in the floor-reared flock was observed starting from day 18. In the E. maxima/*C. perfringens* dual infection model, NE lesions can be produced without mortality in animals reared in wire cages (Williams et al., 2003), while similar models in floor-reared broilers yielded NE-induced mortality ranging from 8 to 12% (Wu et al., 2010; Hofacre et al., 2019).

Natural NE infection induced by reused litter material from a previous flock resulted in mortality from days 15 to 30, ranging from 1.5 to 4.9% across dietary treatments (Palliyeguru et al., 2010). Fernando et al. (2011), by housing birds on wood-shaving litter and removing antibiotics, induced NE infection with mortality of 1.19–1.66% from days 20 to 36. These findings and our observation are consistent with the mortality range reported in subclinical NE affected flocks (Lovland and Kaldhusdal, 2001). Recent work by Calik et al. (2019) and Emami et al. (2019) described a new natural NE disease model by spraying the same coccidiosis vaccine, as used in our study, on litter and feed upon bird placement. An NE outbreak was observed on days 7–9 with overall mortality at around 12%. These findings showed higher mortality which peaked at an earlier age compared to our study but is consistent with our observation that the NE outbreak occurred 1–2 weeks after the coccidial challenge by concentrated Coccivac^®^-B52 vaccine.

### 4.4 Practical Aspects of the Natural Infection Model

Conventional clinical NE models usually involve repeated oral inoculations of coccidial oocysts and *C. perfringens* for consecutive days (Williams et al., 2003; McReynolds et al., 2004; Gholamiandehkordi et al., 2007; Park et al., 2008; Wu et al., 2010; Jayaraman et al., 2013). One of the advantages of the natural NE model is the simplicity of the challenge schedule. Coccidial inoculation typically takes 30 min for two experienced technicians to gavage 100 birds, while feed withdrawal can be done within an hour even in large flocks. Experience from our research facility showed consistent induction of NE across studies using this protocol. Reduced complexity in the challenge schedule can limit animal stress and treatment inconsistency between different personnel, thus contributing to persistent induction of NE disease.

This natural infection model also allows flexibility in designing dietary formulas. The wheat-based broiler chicken diets commonly include xylanase to break down arabinoxylans, decrease viscosity, and increase digestibility in the birds (Lee et al., 2017). Elimination of xylanase has been used to produce natural NE infection (Abildgaard et al., 2010), by increasing feed transit time promoting *C. perfringens* persistence (Choct et al., 2006). Another commonly used dietary predisposing factor, fishmeal, supplies abundant glycine and methionine that enhance *C. perfringens* proliferation and toxin production (Wilkie et al., 2005; Dahiya et al., 2007; Shojadoost et al., 2012). However, the usage of fishmeal is limited in broiler feed due to its high cost and low availability (Frempong et al., 2019). We showed that birds fed with a fishmeal-free diet with xylanase inclusion (flock 3) showed prevalent NE lesions in the gut, suggesting fishmeal inclusion and xylanase elimination are not required for experimental NE induction.

The presence of pathogenic *C. perfringens* is required but insufficient to trigger NE infection. The virulence phenotype of *C. perfringens* is subject to the influence of host epithelium and complex lumen environment (Figure 1). This highlights the cooperative roles of a wide range of environmental factors that contribute to NE development. For research aimed at prophylaxis of NE, it is critical to conduct the evaluation under a condition accurately mimicking the disease development under practical production conditions. The infection model presented in this study was able to reproduce the commonly observed subclinical NE, regarding the severity of symptoms, timing of lesion development, and rate of mortality. By allowing the pathogen to develop *in vivo*, NE researchers will be able to evaluate a prophylactic strategy at an early stage of disease development, when the damage to the animal is most reversible and thus ideal to be targeted. However, more studies are needed to better understand this novel infection approach, such as alterations to mucus characterization, epithelial properties, and immunological function during natural NE induction.

Feed withdrawal introduced on day 18 in this natural NE challenge is important to trigger the timely development of the disease outbreak. At the same time, this approach is believed to cause limited stress to the animals and is considered humane when used properly. Pathogenic *C. perfringens* has a stronger ability in binding extracellular matrices and utilize nutrients released from host intestinal tissue, thus showing a selective survival advantage over nonpathogenic strains during feed withdrawal (Timbermont et al., 2009, 2014; Wade et al., 2015). Feed restriction is often used in conventional NE challenges, not as a designated stressor but as a measure to ensure uniformity of inoculation treatments (Shojadoost et al., 2012). In those situations, the *C. perfringens* inoculation is administered mixed with feed, and feed is usually withdrawn overnight so that the birds will eagerly eat the inoculated feed.

The NE-causing *C. perfringens* are of high diversity in terms of genomic content, with the varied ability to cause intestinal damage. The growing understanding of the differences between strains isolated from animals of different health statuses and geographical regions highlights the need to carefully select appropriate strains to use in experimental NE models. It was reported that 2 *C. perfringens* strains, both isolated from NE-affected chickens and carrying NetB, showed varied virulence and produced different levels of disease severity in experimentally-induced NE (Gharib-Naseri et al., 2019). Knowledge gained using this challenge model can likely be applied across wide geographic regions. The natural, subclinical infection NE challenge model would allow the propagation of pathogenic strains that are locally prevalent. This would be beneficial to the specific region in the understanding of pathogenesis and control strategies against the locally prevalent pathogenic strains.

## 5 Conclusion

The NE infection model presented in this study is based on the natural uptake of *C. perfringens* presented in the housing environment by the chicken. We incorporated multiple NE-associated risk factors to promote the natural development of pathogens, and successfully reproduce subclinical NE. This will contribute to future research aiming at understanding and preventing this disease, by mimicking the natural development of NE in commercial poultry production.

## Data Availability

The original contributions presented in the study are included in the article/Supplementary Material; further inquiries can be directed to the corresponding authors.
